# Development of an integrated cardiac rehabilitation program to improve the adaptation level of patients after acute myocardial infarction

**DOI:** 10.3389/fpubh.2023.1121563

**Published:** 2023-04-17

**Authors:** Xiyi Wang, Li Xu, Geraldine Lee, Antai Song, Jing Shao, Dandan Chen, Hui Zhang, Hanfen Chen

**Affiliations:** ^1^School of Nursing, Shanghai Jiao Tong University, Shanghai, China; ^2^Florence Nightingale Faculty of Nursing, Midwifery and Palliative Care, King’s College London, London, United Kingdom; ^3^Department of Nursing, Renji Hospital, Shanghai Jiao Tong University School of Medicine, Shanghai, China; ^4^School of Nursing, Zhejiang University School of Medicine, Zhejiang, China; ^5^Department of Cardiology, Guizhou Provincial People’s Hospital, Guiyang, Guizhou, China

**Keywords:** cardiac rehabilitation, mHealth, acute myocardial infarction, adaptation, behavior change, intervention mapping

## Abstract

**Background:**

Individual’s adaptation following acute myocardial infarction (AMI) and low attendance of whole-course cardiac rehabilitation (CR) are significant issues. For optimal health post AMI, an integrated CR program aiming at individual’s adaptive behaviors is imperative for improving the CR efficiency and patients’ outcomes. This study aims to develop theory-guided interventions to increase CR attendance and adaptation level of patients post-AMI.

**Methods:**

This study was conducted in a tertiary hospital from July 2021 to September 2022 in Shanghai China. Guided by the theory of adaptation to chronic illness (ACI theory), the study followed the Intervention mapping (IM) framework to develop the interventions for CR program. Four phases included: (1) needs assessment of patients and facilitators using a cross-sectional study and semi-structured, in-depth interviews, (2) identification of implementation outcomes and performance objectives, (3) selection of theoretical methods to explain the mechanism of patients’ adaptive behaviors and to use for behavior change, and (4) development of implementation protocol from the results of the previous phases.

**Results:**

A total of 226 AMI patient-caregivers paired samples were eligible for the data analysis, 30 AMI patients participated in the qualitative inquiry, 16 experts in the CR field evaluated the implementation protocol, and 8 AMI patients commented on the practical interventions. Following the IM framework, an integrated cardiac rehabilitation program using mHealth strategies was developed for AMI patients to facilitate CR attendance and completion, to improve their adaptation level and health outcomes.

**Conclusion:**

Using the IM framework and ACI theory, an integrated CR program was developed to help guide the behavior change and improve adaptation among AMI patients. The preliminary findings suggest that further intervention in enhancing the combination of three-stage CR is required. A feasibility study will be conducted to assess the acceptability and effectiveness of this generated CR intervention.

## Introduction

Cardiac rehabilitation (CR), as 1A-class evidence proposed by multinational guidelines, can improve patients’ cardiopulmonary function and quality of life, and reduce hospital readmission and cardiovascular mortality (1–4). A component of Acute Myocardial Infarction (AMI) treatment is CR program which includes three phases defined as clinical, outpatient, and post-cardiac rehab. According to Million Hearts Cardiac Rehabilitation Collaborative, a 70% participation rate of CR was expected to be achieved by 2022 (5). Analytical data demonstrated a huge gap showing low rates with 24.4% participation rate (7.1% for AMI event) among Medicare beneficiaries in United States (6), 7.05% of AMI patients attending the CR after discharge in China (7), and 1.5% of AMI patients undertaking CR during outpatient treatment in Korea (8). Despite the benefits of CR, CR participation rates remain suboptimal worldwide (4), and thus effective strategies are required (9).

CR is a comprehensive intervention including the optimal use of medication, physical exercise, lifestyle modifications, psychosocial health, and regular follow-up education (10). Outcome measures and performance within CR have been audited in Sweden and United Kingdom demonstrating high-quality CR programs in these countries (10, 11). However, a national survey conducted in 2016 about CR programs in China (12), reported that the estimated prevalence of CR services in China was 24.4% among tertiary hospitals and estimated availability of CR programs was 2.2 per 100 million people. Little is known about the embedding of three CR phases. Redesigning the CR program at an institutional level and improving CR attendance and completion at an individual level has the potential to impact healthcare resources and outcomes and requires further exploration.

Interventions on the different CR phases provide an effective pathway to improve the CR participation and outcomes, but require significant institutional support, multidisciplinary team, and robust referral system for patients (4, 10, 13, 14). Previous studies have demonstrated that CR programs need to be designed and adapted as patient demographics vary and health needs of attendees have expanded across different CR phases (15–17). With current studies mainly focusing on one specific phase of CR, strategies for promoting CR program processes are needed to maintain systematic rehabilitation during health transitions of different contexts. A large-scale trail named Yoga-CaRe program for AMI patients was conducted in India and the United Kingdom to evaluate the effectiveness of four-phase CR program (inpatient care, two-session outpatient care, and long-term home care). The 6 month interventions of Yoga-CaRe program were delivered *via* a booklet, DVD materials, in-person education, and telephonic visits and showed satisfactory results on self-report health and return to pre-infarct activities (18). Evidence for designing and mechanism explanations of transitions among four phases are mainly based on the reviews of literature and consultations of CR experts and Yoga experts lacking patients’ involvement and implementation planning group which are important factors for increasing CR enrollment and completion (9).

Numerous studies showed that mHealth or eHealth strategies using digital technologies were useful in CR programs (19), such as SMS messages, telephonic follow-up (20), social media platforms (21, 22), and smartphone applications (23). As a result of the COVID-19 pandemic, inpatient and centered-based CR programs were limited and inaccessible to patients. Home-based CR has been viewed as a priority to promote an efficient integration in the CR program process with the use of remote monitoring device to support AMI patients (6, 16). Patients’ perceptions of CR and their needs, patients’ preference to mHealth use, experts’ recommendations, and contextual factors are core elements of a personalized CR program, and these challenges need to be considered in the development and implementation of interventions in CR.

Moreover, individual adaptation of a CR program can be a complicated integration of internal and external environment resources and information processing which leads to regain a balance between illness and life (24). Within the CR program, patients have to cope and adapt post AMI (25). The level of adaptation represents the capacity of an AMI patient to address the tasks of rehabilitation, to mitigate the side effects of illness on their lives, and to use the available resources (4, 6). Adaptation is a dynamic and nonlinear process for AMI patients, that is, associated with self-regulation and constant learning to extend the competency of health management (26). It manifests in connections with personal socio-demographics, cognitive processing, relationships with healthcare professionals, and the behavioral reactions to their specific health issues. Investigating adaptation in post AMI patients within CR program with interpersonal-tailored interventions and health behaviors change is needed to determine its efficacy.

Theory-guided implementation is foundational in providing the framework for the development of systematic CR program and for interventional efforts of increasing CR enrolment, adherence, and completion (9). The approach of Intervention mapping (IM) emphasizing an inclusion of a theory basis and evidence has been used to develop, implement, and evaluate interventions about health promotion and behavior change (27, 28). The IM framework which adopts an ecological perspective and system thoughts on the individuals, is consistent with the philosophical statement of adaptation concept describing individuals as adaptive systems. Environmental stimuli (i.e., individual characteristics; and social, cultural, and economical factors) should be assessed before promotion of behavior change. To the best of our knowledge, there are no reported studies using IM framework with the combination of adaptation theory to develop individual-level health interventions likely to improve the rate of CR attendance and completion.

This study aims to describe the development of CR program interventions following the IM framework using patients’ experience of adaptation processing and experts’ recommendations with the objectives of increasing CR attendance and improving individuals’ adaptation level in the post-AMI period. The results from this study could be used to examine and redesign the transitions of cardiac rehabilitation and improve the efficiency of hospital-community-home service in the future.

## Materials and methods

### Study setting and population

This study was conducted in a tertiary hospital from July 2021 to September 2022 in Shanghai China. The CR intervention program was led by an interprofessional research team equipped from medicine, nursing and primary care from universities and the affiliated hospital. The scale of the project was considered regionally within the Shanghai City with a cohort of AMI patients recruited prior to their discharge. This study identified AMI patients who were: (1) aged 18 years or older, (2) diagnosed as first-time AMI and survived, (3) received PCI treatment, and (4) willing to participate and contribute to interventions development. The exclusion criteria were: (1) transition to long-term nursing home and (2) cognitive impairment. The three phases of CR program included in-hospital CR in the cardiac care unit (CCU), center-based CR in the cardiac rehabilitation center (CRC), and center-guided home-bases CR in the patient’s home.

### Ethical statements

The study was approved by the ethics committee of the university affiliated hospital (No. KY2021-098-B). Participants were informed of the study design and provided written consent. Participants were informed that their data were confidential and beneficial to their follow-up rehabilitation. This study adhered to the principles of the Declaration of Helsinki.

### IM phases and methods

**Figure 1:**
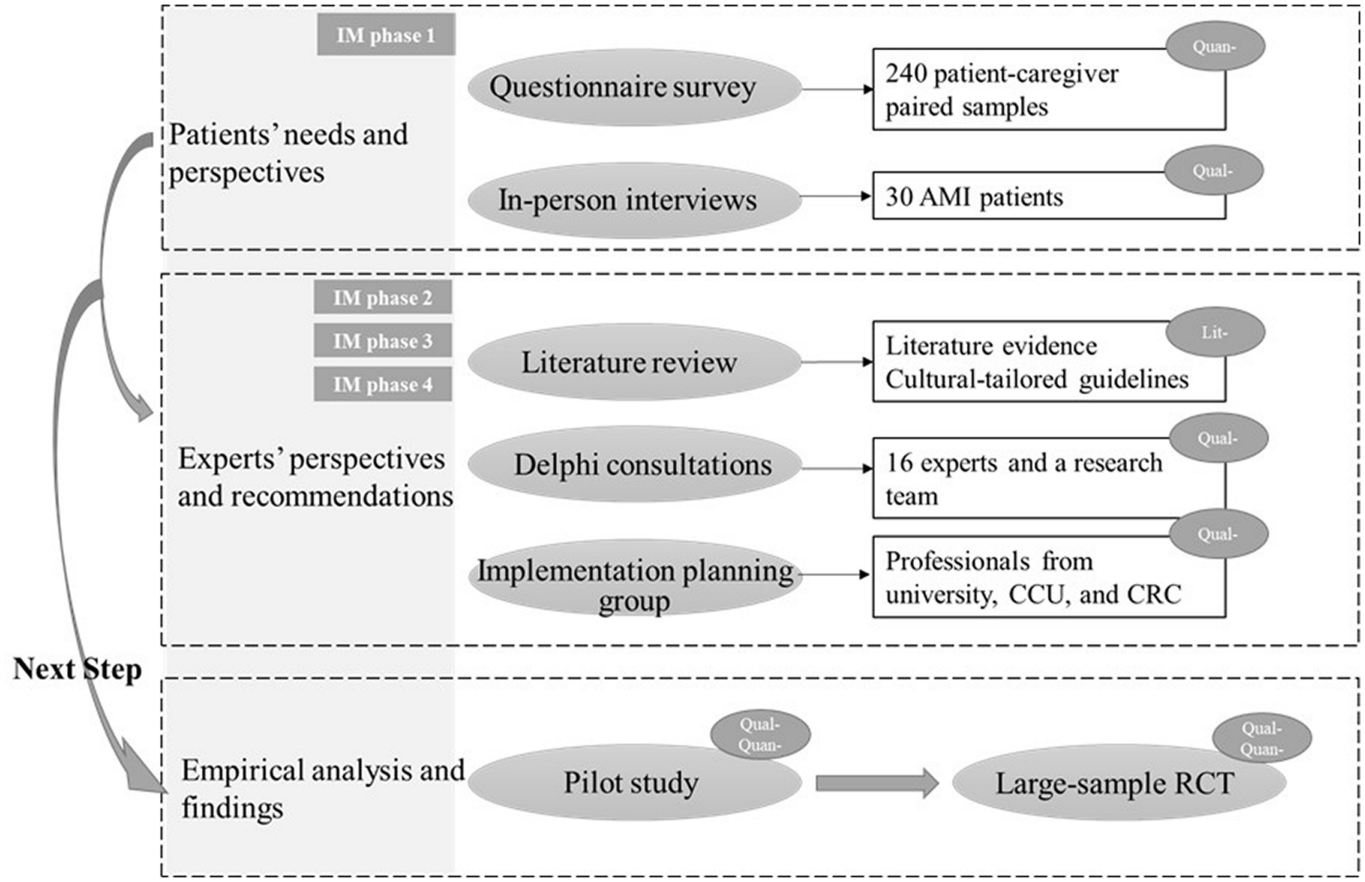
Flow diagram of the research process based on the IM framework.

IM is a robust framework (28), which allows integration of theories and contexts to develop implementation interventions (29), and it was used to guide the study design. To guide our work in the development of CR program, the conceptual framework of Roy’s adaptation theory (24) and middle-range theory of adaptation to chronic illness (ACI theory) (26) were followed, in which adaptation is a process and an outcome of integration of internal and external environments. Thus, we followed the IM framework to develop the CR program and related interventions within the ACI theory. Four phases included (shown in Figure 1), after that, the 5th and 6th phases will proceed at a regional level.

#### Phase one: Needs assessment

The phase of needs assessment comprised four steps.

*Step 1. Establishing an implementing planning group*: core members of the research team liaised with key stakeholders at institutional and individual levels. First, following the standard CR three stages, the contextual factors for intervention implementation were agreed, including the CCU, CRC, home environments, transition measures, and COVID-19 policies. Institutional members involved in CR program were identified and categorized to be CCU group, CRC group, and follow-up group, including physicians and nurses with expertise in cardiac rehabilitation and self-management. Secondly, a purposeful inclusion of experts was undertaken based on personal collaborative network. University-level researchers with expertise in health behavior change and mobile health technology were selected. Two researchers led the 10-member implementation planning group and organized meetings of initial brainstorm and following discussion. Using the approaches of open-ended questions and group voting, qualitative records and quantitative data were analyzed.

*Step 2. Literature reviews*: The needs assessment started with a literature review on an exploration of adaptation in chronic care (30) and electronic health interventions on lifestyle modification (31). Additionally, a scoping review about the coping strategies enacted by cardiac patients was conducted, and with the extraction of influencing factors related to coping capacity (unpublished). Using the keywords of coping and coronary heart disease, English databases Web of Science, PubMed, CINAHL, Cochrane Library, and Embase, and Chinese databases Wan Fang, China National Knowledge Infrastructure, and China Biomed were searched. An initial 2,882 abstracts were screened and 259 full text manuscript were read. According to coping concept defined by Lazarus and Folkman, inclusion criteria were describing coping mechanism and coping behaviors on cardiac outcomes among adult population, primary studies, and English or Chinese language. After excluding reviews, conference abstracts, and other language papers, 18 articles were included for analysis. Moreover, searches of interventional studies that integrated different phases within CR programs and aimed to increase CR attendance or CR completion were made in the initial study. The literature review provided an overview of the core elements related to CR program process at different level of environments (institutional, individual, and interpersonal factors). With a summative review of previous study findings, the attitudes toward general self-management (32) and the adaptive coping strategies of Chinese cardiovascular patients (33, 34) were extracted into the consideration of individual factors.

*Step 3. Assessing patients’ and caregivers’ needs*: From July 2021, a cross-sectional study investigating AMI patients and their caregivers was conducted using a convenient sample in accordance with the Strengthening the Reporting of Observational Studies in Epidemiology (STROBE) Statement. This part of study was to assess patients’ and caregivers’ readiness to return home from hospital following acute care hospitalization. In addition to collecting patients’ socio-demographic information, valid and culture-adapted scales with authors’ permissions were used, including Scale of Systemic Family Dynamics (SSFD) (35), Readiness for Hospital Discharge Scale (RHDS) (36), and Quality of Discharge Teaching Scale (QDTS) (37). Bedside nurses at CCU were trained to collect the questionnaire data. Finally, 240 patient-caregiver paired samples were enrolled for the survey of the discharge readiness, determinants of adaptation to illness status, and perceived family function (unpublished results).

*Step 4. Exploring patients’ perceptions and experiences of CR*: To have a better understanding of the factors influencing the partial or full implementation of this CR program, sub-sequential qualitative data collected from interviews were made by two principal researchers (one was PhD qualified researcher and expert in qualitative study; the other was clinical nursing specialist with abundant experience). From the perspectives of the patients, we explored patients’ adaptive coping strategies for long-term CR and needs related to CR program (i.e., mobile health experiences and challenges, acceptance of clinic follow-up alternatives). Additionally, the barriers and facilitators to implementation of integrated interventions were elucidated. Participants who met the inclusion criteria of adaptive coping (defined by hospital-based CR attendance rate, regular clinical visits, and meeting goals of guideline-recommended CR targets and levels) were approached and recruited. Using a convenient sample, 30 AMI patients were recruited by a CCU nurse and a CRC therapist. The interview guides were developed according to the findings of *step 3* and the clinical experience of the researchers. Each participant participated in a face-to-face audiotaped interview lasting approximately 40–80 min. Eleven AMI patients expressed their attitudes and motivation toward CR program in hospital pre-discharge and 19 AMI patients who achieved their goal of centered-based CR shared their experiences of developing adaptive coping strategies (unpublished). The study design was performed aligning with the standards for Reporting Qualitative Research (SRQR).

#### Phase two: Identification of implementation outcomes and performance objectives

Based on the logical model of needs assessment (Phase one), we decided to formulate program streams for patients and CR implementation separately following the culturally tailored guidelines and using the approach of the research team. The goals of the program would not only focus on promoting the integration of internal and external environments of an individual, but also on bringing AMI patients in contact with healthcare professionals thereby extending their usage of CR programs and their capacity of adaptation. Seven researchers with expertise in clinical practice, interventional studies, and theory-testing research within the field of cardiovascular had discussions on performance objectives at the practical and theoretical level. With the findings of need assessment, the performance objectives were adjusted. The general performance objectives would be evaluated by improved capacity of adaptation processing and better quality of life.

#### Phase three: Selection of theoretical methods

Concept of adaptation demonstrates the idea of philosophical states describing the relationship of the person and environment (24). Using the conceptual-theoretical-empirical framework (38), the process of adaptation was deconstructed based on the empirical evidence. Within the context of chronic health conditions, ACI theory describing the mechanism of patients’ adaptive behaviors (26) was used to guide selection of the theoretical methods for developing interventions belonging to this integrated CR program. Additionally, corresponding to KoK’ taxonomy (39), selection of behavioral change methods for our CR program was performed by reviewing the relevant literature. Referring to practical applications (39) (herein for practice use in ways that fit the AMI patients and the context in CCU and CRC), inductive analysis was performed based on patients’ experience and perspectives to condense the meaning of theoretical methods. Discussions with the research team took place for clarification of the theoretical methods and practical applications and all queries resolved.

#### Phase four: Development of implementation protocol

Chinese guidelines of cardiac rehabilitation with cultural adaptation of the guidelines from ESC, AHA, and ACC were followed and reviewed several times considering the real-world context. Completion of the above phases, implementation planning group and implementors from CRC had a detailed discussion about the practical applications and intervention delivery. Iterative updates on the list of behavior change methods and practical applications were embedded. Meanwhile, all relevant materials were reviewed again by two principal researchers and synthetized as initial version of implementation protocol, then intervention contents were sent to 16 experts with different academic backgrounds within the cardiovascular field *via* email and instant communication application. The Delphi expert panel consisted of cardiac specialist physicians, cardiac specialist nurses, behavioral intervention experts, physical rehabilitation therapists, and methodological experts (40). All experts reviewed and evaluated the feasibility of the intervention procedures and corresponding outcome indexes independently. Based on experts’ feedback, we revised the intervention content and confirmed the content with the group. Apart from measuring adaptive capacity and discharge readiness at baseline, process indicators related to health transitions were included (i.e., each phase of CR enrollment rate and attendance rate, and follow-up frequency) for evaluating the adaptation processing. What’s more, meetings to discuss the interactions of remote device use and patients’ data feedback were held. Further modifications of implementation protocol were made and supported by technological companies. Eight AMI patients were invited to comment on the implementation protocol for the sub-sequential trial study. The implementation protocol was then revised (intervention change components, behavior change methods, and practical applications).

### Data collection

Mixed-method approaches were performed and varied in different study stages according to the research questions (see Figure 1). All involved investigators were trained for different parts of study accordingly. Within the IM framework and ACI theory, integration was made by combining quantitative and qualitative data. Quantitative data were collected from a cross-sectional study and other statistic-based judgments by trained clinical nurses. Qualitative data were original from semi-structured interviews for describing patients’ experiences and exploring individual’s perspective on why and what interventions should be included and how implementation should be conducted. Additionally, integration of cultural specificity and evidence translations for real-world practices were considered using implementation planning group and Delphi experts.

### Data analysis and synthesis

The raw data were transferred into digital version by trained nursing students. The collected data were managed using Excel spreadsheet and Word file. For the rigor of quantitative data, clinical nurses checked the questionnaires, and a well-trained nurse intern undertook a check of the digital data. The sample size was estimated using the G-power 3.1 and for the next-step empirical study. According to the data from a 6-month behavior change program for heart failure patients, an effect size (Cohen’s *d* = 0.54, *r* = 0.26) was determined by the difference in scores of adaptation evaluation (38). The sample size of 182 participants would be adequate to achieve a 95% power at 0.54 effect size at a 5% level of significance with the T-test method. SPSS 28.0 software was used to calculate for a baseline description of patients’ needs. All raw scores of scales were transferred into standard scores to make comparations. Descriptive analysis was performed to determine the mean and standard deviation (SD) for continuous variables and frequency/percentages for categorical variables. Regarding the qualitative data, all interviews and group discussions were recoded using a professional recording device and transcribed and reviewed, and then analyzed *via* the NVivo 12.0 plus software. The themes of the interview were used as coding categories under the umbrella of theory concepts with a deductive view. Meanwhile, an inductive content analysis was performed to compensate patients’ feelings, perceptions, and expectations in the perspectives of intervention implementation.

## Results

This section describes the results from the phases synthesized using the results of needs assessment, implementation outcomes and behavior objectives, the theoretical methods and strategies, and implementation protocol.

A total of 226 patient-caregiver paired samples were analyzed. They were mean age 62.09 years (SD 12.57) male (80.5%), Han nationality (97.8%), married (88.1%), no religion (86.7%), retired status (58.0%), and with health insurance (81.0%). Among 226 caregivers, the relationships with AMI patients varied with 48.7% couples, 45.10% parents and children, 3.5% siblings, 1.3% grandparents and grandsons, and 1.3% friends.

### Findings for needs assessment

**Figure 2:**
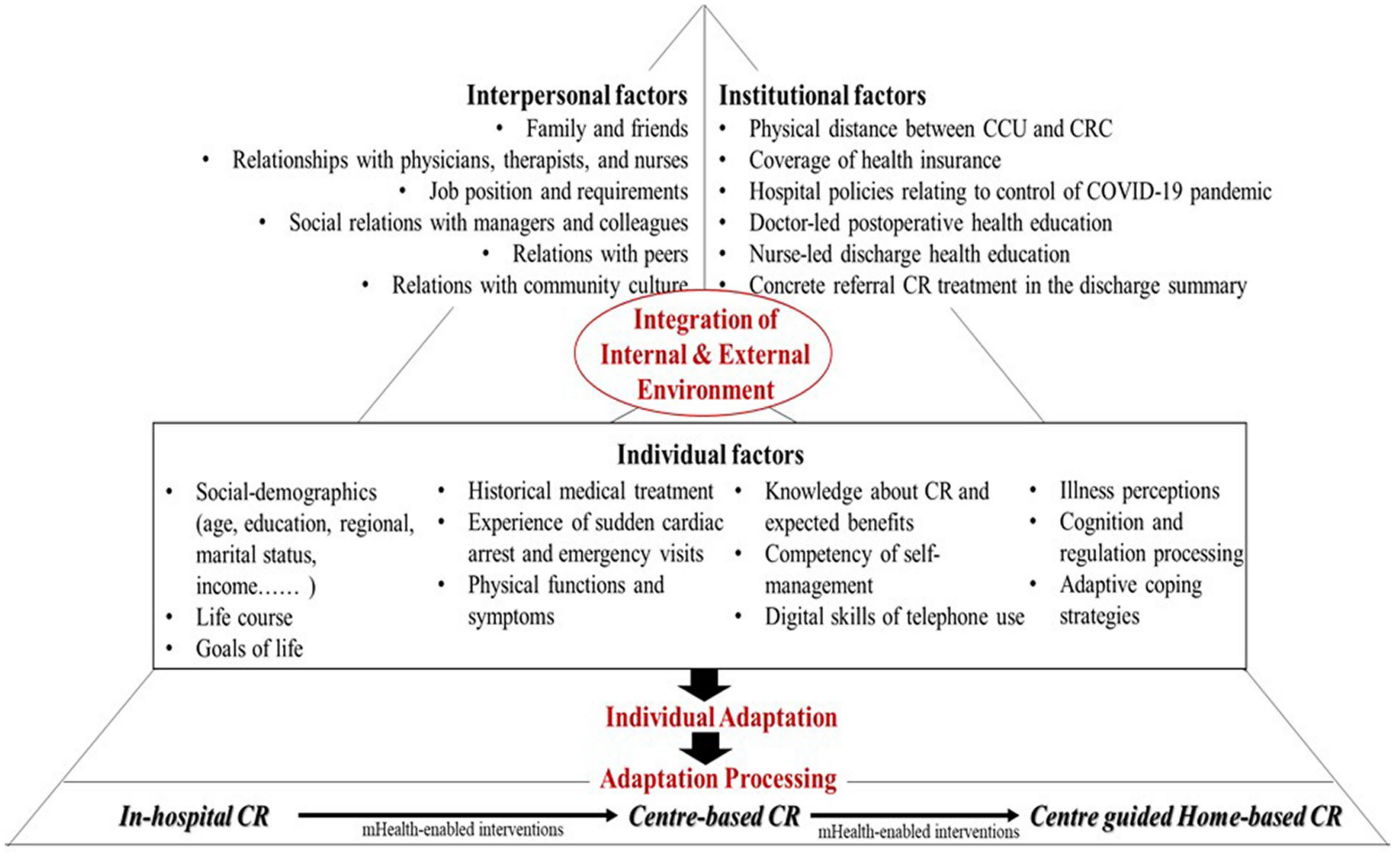
The logic model describing the institutional, interpersonal, and individual determinants for CR health behaviors.

All members in the implementation planning group reached an agreement that a well-designed, patient-centered, caregiver-involved, and multidisciplinary-led CR program should be developed to improve the attendance and completion of CR program. Figure 2 demonstrates the determinants of CR behaviors of AMI patients and conceptualizes a logic model of individual adaptation for potential interventions to focus on and then to address patients’ needs, which were derived from theory concepts, empirical evidence, and practical knowledge. This framework proposes a statement that incorporates institutional, interpersonal, and individual factors that includes different levels of resources and that will impact on AMI patient’s cognition and regulation. These components will then lead to the output of behaviors and changes in health outcomes.

*Institutional factors*. Combining literature review findings and previous results from a retrospective study investigating the CR attendance (7), institutional factors were identified. These included physical distance between CCU and CRC, coverage of health insurance, hospital policies relating to control of COVID-19 pandemic, doctor-led postoperative health education, nurse-led discharge health education, and concrete referral CR treatment in the discharge summary.

*Individual factors*. As shown in Table 1, the assessment of discharge readiness of patients and caregivers was performed in the time point of transition from the in-hospital CR to center-based CR. Findings from quantitative data showed that patients and their caregivers with different social demographics, history medical treatment, and physical symptom resulted in varied discharge readiness, and AMI patients reported sub-optimal patient education discharge information. Findings from the sub-sequential qualitative interviews revealed that AMI patients demonstrated a willingness to attend CR programs. With clear life goals and motivations for behavior change. For example, an AMI patient a retired policeman who attended centered-based CR, stated his goal of regaining adequate energy level to enjoy life not for thorough rehabilitation: “*I would follow your therapy planning and come here regularly but would not adhere to the intensity of therapy strictly. I hope I can feel happy when I attend the CR program*.” On the other hand, younger AMI patients tended to search for more CR knowledge and think about the CR benefits. The competency of self-management and digital skills affect patients’ decisions to accept CR referral. Additionally, previous studies provided an insight regarding coping mechanisms with three core elements of illness perceptions, cognition and regulation processing, and adaptive coping strategies (34). These factors have also been observed among patients in the post-AMI period.

**Table 1:** Differences in AMI patients’ and caregivers’ perceived discharge readiness and family dynamic (*n* = 226).

Assessment	Patient (*n* = 226)	Caregivers (*n* = 226)	*T* value	Two-side *p*
SSFD-FA	80.79 ± 16.18	70.23 ± 11.40	−3.490	<0.001
SSFD-IN	73.73 ± 17.63	84.31 ± 15.88	22.667	<0.001
SSFD-SL	33.12 ± 22.55	35.00 ± 24.29	−26.065	<0.001
SSFD-IC	75.06 ± 19.78	79.46 ± 16.83	3.287	0.001
Total RHDS	82.70 ± 13.49	77.57 ± 16.11	3.913	<0.001
RHDS- personal status	79.96 ± 16.36	74.23 ± 17.34	3.867	<0.001
RHDS- coping ability	82.83 ± 15.46	75.58 ± 24.26	4.032	0.504
RHDS- expected support	84.59 ± 14.53	83.41 ± 18.11	0.669	<0.001

Maximum score was 100 (using the method of standard score).

SSFD, Scale of Systemic Family Dynamics; FA, family atmosphere; IN, individualization; SL, system logic; IC, illness concept; RHDS. Readiness for Hospital Discharge Scale.

*Interpersonal factors*. We tested the differences on patients’ and caregivers’ perceived discharge readiness and perceived family dynamic using the method of paired sample T-test. The result showed that all subscale of SSFD and RHDS (expect for the coping ability) had statistically significant differences between patients and caregivers (*p* < 0.001). Comparing with the caregivers, AMI patients perceived lower level of family function and better readiness for discharge (see Table 1). The perceptions of discharge readiness and family dynamic were further explained by qualitative interviews. All AMI patients thought highly about the functions of their family caregivers in the process of CR programs. Furthermore, six factors related to interpersonal relationships were proposed from the aspects of family members, friends, healthcare providers, work colleagues, other peers, and community.

### Findings for implementation outcomes and performance objectives

**Figure 3:**
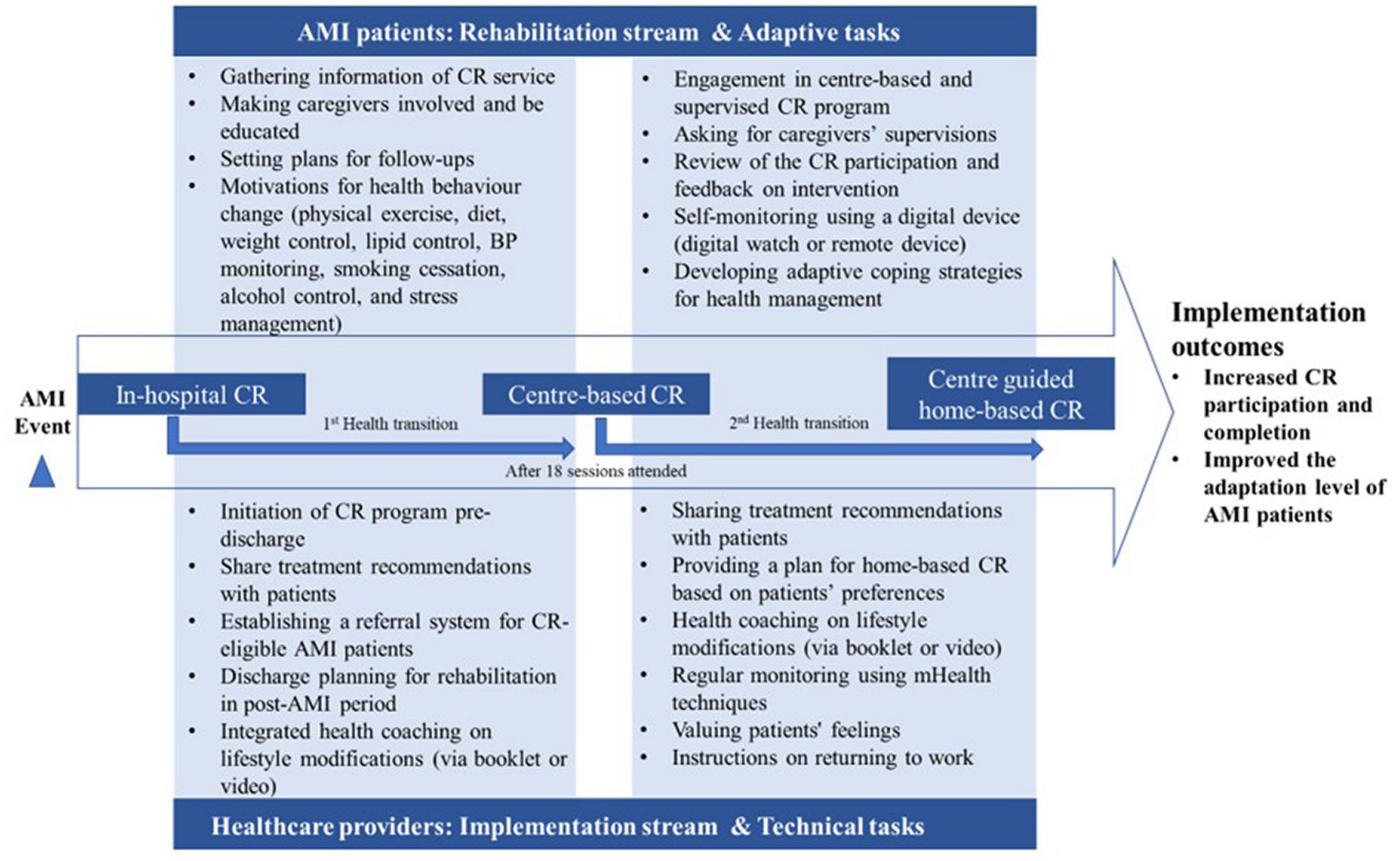
The integrated flowchart with three-phase performance objectives.

The combined findings of needs assessment, culture-adapted guidelines, and clinical experiences of implementation planning group, implementation outcomes, and performance objectives were synthesized (see Figure 3). Chinese guidelines of CR and secondary preventions of cardiovascular diseases were reviewed, core components of CR program were extracted, including initiation of CR program in hospital, systematic individual patient assessment, personalized CR therapy (physical exercise, diet, weight control, lipid control, BP monitoring, smoking cessation, alcohol control, and stress management), and managerial strategies for CR programs. Moreover, the performance objectives in the health transition linking sub-sequential phase of CR program were explored based on the analysis of patients’ expectations and their self-initiated coping behaviors. For instance, a shipyard worker appeared to complain that “*I read the doctor’s discharging summary, then I know I should start center-based CR in one month after hospital discharge……Now because I was required to return to work, I would like to know how to transfer to home rehabilitation. Sometimes, I would send messages to the therapist using my private relationship (he privately contacted his doctors)*.” Specifically, two streams of healthcare providers and patients were outlined, accordingly adaptive tasks and technical tasks as the determinants of implementation intervention were proposed (see Figure 3).

### Findings for theoretical methods

**Figure 4:**
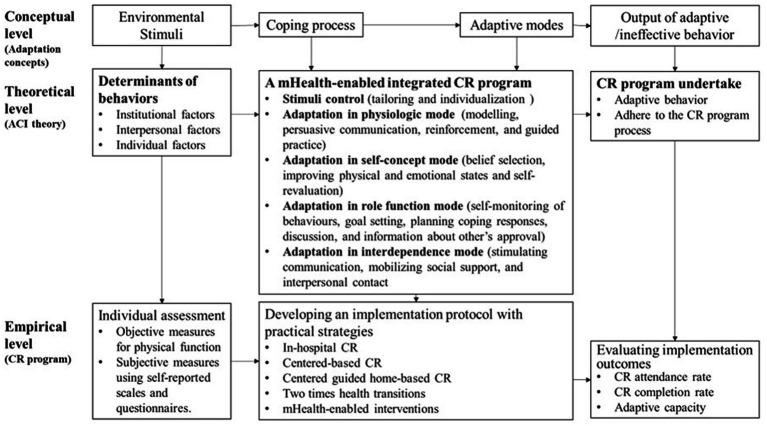
Conceptual-theoretical-empirical framework for this integrated CR program based on the ACI theory.

Using deductive analysis of the ACI theory, the theoretical methods for this CR program were tabulated with a combination of behavior change techniques. Figure 4 illustrates how implementation interventions work on CR attendance and completion through their impact on the determinants and behaviors of those responsible for CR program adoption under the umbrella of theory basis. Additionally, these provided information for general intervention tasks by control of stimuli (details as needs assessment at the institutional, interpersonal, and individual levels) and promotion of coping process in terms of physiological mode, self-concept mode, role function mode, and interdependence mode (details as performance objectives for adoption, implementation, and evaluation).

### Findings for the implementation intervention components

In this study, the CR program will be implemented in a qualified CRC equipped with a standard therapy pattern for three CR phases. Aligning with the ACI theory and evidence-based CR evaluation, the implementation outcomes for measuring adaptation level will target CR attendance rate, CR completion rate, and adaptive capacity (see Figure 4). Implementation interventions, consisting of three CR phases, general objectives, theoretical methods, practical applications, and delivery methods, were integrated based on previous studies and are presented in Table 2. Patients’ health needs and perspectives extracted from qualitative and quantitative data were analyzed to be facilitators and barriers of CR attendance as the determinants of CR behaviors. These findings from IM phase one guided the identification of performance objectives created in IM phase two. After reviewing the ACI theory, which unite all concepts throughout the whole project, the conceptual-theoretical-empirical framework for practice guide was developed and behavior change methods were selected and confirmed according to experts’ perspective and recommendations. The theoretical methods created in IM phase three, laid the foundation for implementation interventions. Additionally, patients’ comments on the practical interventions were embedded into the implementation protocol. For example, 3 AMI patients who were IT engineers expressed their expectations on how doctors and nurses could help them with self-monitoring. “*I designed a digital and renewable chart to track my daily routine. It would be great if you can give me feedback every week.”* Therefore, the format of discussion and care delivery modes were enriched and individualized to realize the goal-setting and planning coping response. Overall, Table 2 showed the mHealth-enabled integrated CR program which was combined with experts’ suggestions from the Delphi panel and patients’ comments.

**Table 2:** Implementation intervention plan for the integrated cardiac rehabilitation program.

Phase of CR program	General objectives	Theoretical methods	Practical applications	Delivery methods
In-hospital CR	Participants are informed of CR program pre-discharge. Participants have an improved motivation to attend CR programs. HCPs share treatment recommendations with patients and caregivers. HCPs provide health coaching on lifestyle modifications.	Tailoring and individualization	Participants are asked to watching video and booklet related to knowledge of heart diseases and cardiac rehabilitation. Each participant will take part in the bedside physical activity under the supervision of nurses and therapists. Nurse-led health coaching will be held to educate patients and caregivers about the knowledge and skills of lifestyle modifications after discharge.	Face-to-face supervisions Digital information WeChat communication
		Persuasive communication and discussion	Physician will share treatment recommendations and refer CR program to eligible patients when making an individualized follow-up plan. Participants receive information of CR program structure at discharge consultation from nurses. Participants are able to discuss with HCPs to set a detail follow-up plan.	Face-to-face supervisions
Center-based CR	Participants regularly attend CR program and actively communicate with HCPs. Participants are better able to share their experiences of coping responses. Participants positively report their feedbacks regardless of interventions. Participants can use the WeChat platform for a better interaction with HCPs.	Modeling	Participants understand the challenges caused by the illness and learn to regain the control of life.	Face-to-face supervisions
		Planning coping responses	Participants are informed of potential adaptive tasks in the period of CR program and transition to home care. Participants are encouraged to develop adaptive coping strategies using personal resources.	Face-to-face supervisions Telephone follow-ups
		Tailoring	Physician will provide suggestions on how to perform regular exercise-dominated center-based CR program. Cardiac specialist nurses will provide an individual assessment of physical functions. Therapist will provide a baseline assessment of patients’ exercise capacity. HCPs provide participants with the details relating to intensity of individual-level CR program (i.e., length, time, and methods). Participant will be trained to use WeChat platform and hospital website to access health information.	Face-to-face supervisions WeChat communication
		Belief selection and improving physical and emotional states	Participants are encouraged to talk about their physical and emotional feelings. Participants learn to find something positive in the crisis events and to establish a positive belief of health management over time. HCPs will help with evaluate their coping resources and give suggestions accordingly.	Face-to-face supervisions Telephone follow-ups
		Stimulating communication, mobilizing social support, and interpersonal contact	Participants are encouraged to discuss their true feelings with their caregivers in daily life and HCPs in clinic follow-up. Participants are encouraged to tell HCPs their needs and puzzles whatever they came across the rehabilitation process. Participants can communicate with peers in CRC clinic visit and share experiences with each other.	Face-to-face supervisions WeChat communication
		Self-evaluation and reinforcement	After each session, participants will be invited to give feedbacks on their perceived promotions of physical functions and emotional feelings. Participants will be informed of which ways are better based on their past experiences and with evidence support.	Face-to-face supervisions Telephone follow-ups
		Goal setting and information about other’s approval	Every week, participants will be informed of their weekly goals and potential goals for in-period home life. HCPs discuss with patients on their initiated attitudes, knowledge, personal habits, and behaviors toward self-management in post-AMI period.	Face-to-face supervisions WeChat platform
Centre-guided home-based CR	Participants use digital techniques to track exercise diary and monitor their heart functions. Participants develop their adaptive coping strategies for long-term health management. Participants get improved capacities of health management by attending and completing CR programs.	Guided practice	Participants will be trained to use alternatives of unhealthy dietary ingredients and available equipment to establish new habits at home with the guidance of therapists and nurses. Participants will follow the educational videos to do physical exercise at home. Participants will be trained to use remote digital monitoring device when doing exercises, hanging out, or traveling to make themselves safety.	Face-to-face supervisions WeChat communication Remote digital monitoring device
		Goal setting and planning coping responses	Participant will be phoned every week to renew their goals of behavior change. Participant are encouraged to use the problem-focused coping strategies and report their successful experience of forming a new healthy behavior.	Telephone follow-ups WeChat communication
		Self-monitoring of behaviors	Participants will be suggested to keep a record of lifestyle behaviors by choosing a preferable approach (i.e., digital diary, physical paper diary, and WeChat records). Participants will be asked their behaviors of diets, physical activities, clinic visits, stress management, medicine undertake, smoking cession, and alcohol drinking.	Face-to-face supervisions A telephone-transmitted EKG record Real time physical data collection
		Discussion and information about other’s approval	Participants are encouraged to bring their health diaries and to report to HCPs in clinic follow-up for efficient discussions. Participants can get personalized answers and information related to their questions from HCPs because HCPs know their background well.	Face-to-face supervisions Telephone follow-ups WeChat communication
		Stimulating communication, mobilizing social support, and interpersonal contact	Participants are encouraged to go outsides and socialize with others. An action plan for returning to work will be drawn up for eligible participants with significant others’ opinions.	Face-to-face supervisions Telephone follow-ups WeChat communication

HCP, Healthcare providers.

## Discussion

This study provides an example of using the IM framework with a combination of ACI theory and evidence of cardiac rehabilitation to create complex interventions to support the improvement of CR attendance and completion. Following the IM framework, implementation planning group participated in each phase of study development which strengthened the practical applications for real-world interventions. Moreover, AMI patients with realistic experiences from different CR phases gave abundant sources of perspectives on intervention development and also on how they should be implemented. The findings demonstrated that facilitation of individual adaptation in the post-AMI period needs to consider as early as possible before hospital discharge. Patients’ and caregivers’ needs associated with long-term cardiac rehabilitation are the foundation of behavior change interventions. Our study provided an opportunity for health professionals to better integrate three CR phases based on contextual resources to make prosperous feasibility for patients CR undertake (41).

To improve compliance with CR program, CR knowledge and CR service should be recommended to AMI patients formally in the hospital stay. We found that the Chinese population had a good level of knowledge about minimizing physical activity in the immediate period after an illness. Therefore, there is a need to change this belief post AMI as the initiation of CR program is important. Persuasive communication and discussion aiming at introducing CR program could be regarded as educational strategies and be held by an interprofessional team. Meanwhile, methods of tailoring a CR program and individualizing it could be encouraged. This approach is in line with a previous study showed that participants in an educational program would be engaged actively if the intervention contents were tailored according to their needs (42). Thus, individuals’ needs assessment should include a better connection and transition to next CR phase in outpatient setting.

We found that physicians’ attitudes toward CR therapy and therapists’ involvement in bedside CR played an important role in center-based CR attendance. Findings of discharge readiness assessment showed that AMI patients and caregivers had high-level needs of health coaching on lifestyle modifications and long-term follow-up. Also, patients were strongly influenced by health professionals who recommended a CR program. The phenomenon may be attributable to an improvement of professional training for educators within the CR field. Similar to other CR programs (43), an individual evaluation of an individual patients socio-demographics, level of functionality, and medication treatment are required. Moreover, combining an adapted behavior change method and performance objectives for adaptation improvement has been observed in several studies (44, 45). Due to the essence of ACI theory explaining the coping process and combined with the empirical data from patients and caregiver, we used the approach of modeling and planning coping response to help with the development of patients’ adaptive behaviors. It is acknowledged that patients can actively learn and find a suitable way with a guidance of healthcare professionals, ultimately to adapt to changeable circumstances in a crisis. According to a mixed-method study on women’s adjustment to AMI (46), coping strategies, self-efficacy, social support, and quality of life were core elements and should be considered. Similarly, the factors mentioned above were embedded and addressed by combination use of behavior change methods. In future studies, participatory problem-solving and planning method can be taken into consideration in clinical practice for a better connection of center-based CR and center-guided home-based CR.

Regarding the inventions for center-guided home-based CR, we found that it takes time for patients to step back into a normal life. In this transition period, interventions on rebuilding habits of health behaviors and improving the adaptive capacity are needed. Aligning with the performance objectives, nine interventional methods were identified to perform CR at home, including guided practice, goal setting, planning coping responses, self-monitoring of behaviors, discussion, information about other’s approval, stimulating communication, mobilizing social support, and interpersonal contact. The findings are supported by previous studies where interventional strategies of peer support and reflection discussion were used in a community-informed virtual world-based CR program (47). A systematic review (48) showed that methods of social support and goal setting were frequently adopted in the home-based CR program. In this process, considering the convenience of intervention delivery, mobile technologies for enhancing the interactions of information, and communication appeared to offer appropriate options for bridging the gap over different phases of CR involvement. Telephone contact and instant message application provided opportunities for AMI patients to keep in touch with healthcare professionals. Remote coaching with indirect exercise supervision *via* digital device also is commonly used (13). However, more explorations on integrating mobile techniques and implementation inventions for use in the clinical contexts are needed in future studies.

### Next steps for evaluating implementation protocol

With the guidance of the IM framework, we have identified the core components of implementation interventions for practical use to facilitate individuals’ adaptive behaviors to CR therapy. Additionally, digital strategies based on current mobile technologies are selected and attached to different CR phases. For better implementation in subsequent trials, we will review patients’ perspectives and feedback through a thematic analysis with all the results and make the intervention practices. Meanwhile, we will include software engineers as part of our research team and design a smartphone application. The features of planned application will be based on intervention contents developed in this study. Then, pilot studies and a randomized controlled trial will be conducted to evaluate the feasibility, effects, and acceptability of the CR program. Underpinning by the theory basis, this lends itself to guide the analysis of the interactions among different implementation interventions and makes it possible to determine how improvements in one component may influence the function of another.

## Conclusion

This study adopted a robust framework to develop interventions for using in a mHealth-enabled integrated CR program underpinned by the adaptation theory to improve the CR attendance and CR completion of AMI patients. Empirical evidence shows the values of IM framework and ACI theory for use in real-world practice. Future studies can focus on testing the effectiveness of implementation interventions on increasing the CR undertake and whether is accepted by AMI patients and beneficial to their adaptation, ultimately to improve individual-level health outcomes.

## Data availability statement

The original contributions presented in the study are included in the article/supplementary material, further inquiries can be directed to the corresponding author/s.

## Ethics statement

The studies involving human participants were reviewed and approved by Ethics committee of the Renji Hospital (No. KY2021-098-B). The patients/participants provided their written informed consent to participate in this study.

## Author contributions

XW conceptualized the study, carried out the study, and led the manuscript writing. LX carried out the study and was responsible for the clinical data collection. GL reviewed and revised the manuscript. AS and HC collected the data and helped sort out the research materials. JS, DC, and HZ were the members of research team and assisted with study development. All authors contributed to the article and approved the submitted version.

## Funding

This study was supported by Shanghai Sailing Program (21YF1422400), Shanghai Jiao Tong University School of Medicine: Nursing Development Program, KC Wong Fellowship, and the Innovation research team of high-level local universities in Shanghai (SHSMU-ZDCX20212801). The findings and opinions expressed in this paper are those of the authors and do not reflect the official position of funders.

## Conflict of interest

The authors declare that the research was conducted in the absence of any commercial or financial relationships that could be construed as a potential conflict of interest.

## Publisher’s note

All claims expressed in this article are solely those of the authors and do not necessarily represent those of their affiliated organizations, or those of the publisher, the editors and the reviewers. Any product that may be evaluated in this article, or claim that may be made by its manufacturer, is not guaranteed or endorsed by the publisher.
